# De novo transcriptome analysis of *Cnidium monnieri* (L.) Cuss and detection of genes related to coumarin biosynthesis

**DOI:** 10.7717/peerj.10157

**Published:** 2020-11-06

**Authors:** Yuanyuan Shi, Shengxiang Zhang, Daiyin Peng, Chunmiao Shan, Liqiang Zhao, Bin Wang, Jiawen Wu

**Affiliations:** 1 Anhui University of Chinese Medicine and Anhui Academy of Chinese Medicine, Hefei, China; 2 Key Laboratory of Xin’an Medicine, Ministry of Education, Anhui University of Chinese Medicine, Hefei, China; 3 Synergetic Innovation Center of Anhui Authentic Chinese Medicine Quality Improvement, Hefei, China

**Keywords:** *Cnidium monnieri* (L.) Cuss, Coumarin, RNA-Seq, Transcriptome, Gene expression, Metabolic pathways

## Abstract

*Cnidium monnieri* (L.) Cuss (*C. monnieri*) is one of the most widely used traditional herbal medicines, exhibiting a wide range of pharmacological functions for treating asynodia, trichomonas vaginitis, and osphyalgia. Its important medicinal value comes from its abundance of coumarins. To identify genes involved in coumarin biosynthesis and accumulation, we analyzed transcriptome data from flower, leaf, root and stem tissues of *C. monnieri*. A total of 173,938 unigenes with a mean length of 1,272 bp, GC content of 38.79%, and N50 length of 2,121 bp were assembled using the Trinity program. Of these, 119,177 unigenes were annotated in public databases. We identified differentially expressed genes (DEGs) based on expression profile analysis. These DEGs exhibited higher expression levels in flower tissue than in leaf, stem or root tissues. We identified and analyzed numerous genes encoding enzymes involved in coumarin biosynthesis, and verified genes encoding key enzymes using quantitative real-time PCR. Our transcriptome data will make great contributions to research on *C. monnieri* and provide clues for identifying candidate genes involved in coumarin metabolic pathways.

## Introduction

*Cnidium monnieri* (L.) Cuss, an annual plant of the Umbelliferae family native to China and Vietnam, exhibits a diverse set of pharmacological activities including anti-osteoporotic (An et al., 2016), anti-adipogenic (Shin et al., 2010) and anti-fungal properties (Wang et al., 2009). Many chemical compounds have been isolated and purified from *C. monnieri*, including coumarins, volatile oils, chromones, triterpenoids, glycosides and glucides (Li et al., 2015). Coumarins are the most abundant compounds in *C. monnieri*, of which osthole is considered the dominant chemical constituent with a broad range of anti-bacterial, anti-hepatitis and anti-tumor effects (Cai et al., 2000; Liu et al., 1999; Wu et al., 2017).

Coumarins are a large class of natural products found in higher plants (Venugopala, Rashmi & Odhav, 2013). They have attracted a great deal of interest due to a wide range of pharmacological activities including anti-inflammatory, anti-diabetic and anti-tumor functions (Chong et al., 2002). Coumarins originate from the phenylpropanoid pathway and are subclassified into simple coumarins, furanocoumarins, pyrano-coumarins and others (Yang et al., 2015). Simple coumarins include osthole, scopoletin, umbelliferone and esculetin (Bourgaud et al., 2006). The biosynthesis of coumarins starts with the formation of phenylalanine (Kosuke et al., 2006). Phenylalanine ammonia-lyase (PAL) catalyzes phenylalanine transformation into trans-cinnamic acid; the conversion of trans-coumaric acid to p-coumaroyl-CoA is then catalyzed by cinnamate 4 hydroxylase (C4H) and 4-coumarate-CoA ligase (4CL); p-coumaroyl-CoA is further converted to p-coumaroylshikimic acid and 2,4-dihydroxycinnamoyl-CoA catalyzed by shikimate hydroxycinnamoyl transferase (HCT) and trans-4-coumaroyl-CoA 2-hydroxylase (C2′H), respectively; the 2,4-dihydroxycinnamoyl-CoA and p-coumaroylshikimic acid undergo further reactions to produce various coumarins (Kai et al., 2008; Vialart et al., 2012). Although, the enzymes participating in each step of coumarin biosynthesis have been determined, information regarding the genes encoding these enzymes is still insufficient.

RNA-sequencing (RNA-Seq) has been widely applied as a powerful method for discovering and predicting functional genes, and revealing gene expression patterns, especially in non-model species for which reference genome sequences are insufficient (Chu & Corey, 2012). To date, dozens of medicinal plants have been subjected to RNA-Seq analysis, including recent transcriptome analyses of *Melilotus albus* (Luo et al., 2017), *Artemisia argyi* (Liu et al., 2017), *Arisaema heterophyllum* (Wang et al., 2018), *Clinopodium chinense* (Shi et al., 2019) and *Polygonatum cyrtonema* (Wang et al., 2019). However, such analytical approaches have not been used to obtain comprehensive transcriptomic resources for *C. monnieri*.

The purpose of this study was to uncover functional genes associated with or playing regulatory roles in coumarin biosynthesis. We conducted transcriptome sequencing and gene expression profiling in *C. monnieri* using BGI-500 sequencing technology. Numerous potential genes and differentially expressed genes (DEGs) involved in coumarin biosynthesis were screened using *de novo* transcriptome sequencing. Our findings will increase our understanding of the molecular mechanisms of coumarin biosynthesis in *C. monnieri* and provide clues for exploring functional genes in other plants having close relationships with *C. monnieri*.

## Materials and Methods

### Sample preparation and RNA extraction

Whole *C. monnieri* plants were collected from Tongcheng city in Anhui Province, China in May 2018, with verbal permission of the Manager Zhongquan Fang (Anhui Bowen agriculture and Forestry Development Co., Ltd), and authenticated by Professor Dequn Wang (Anhui University of Chinese Medicine). Prior to the experiment, the plants were grown at a temperature of 22−26 °C/14−18 °C (day/night) and relative humidity of 65–80%. All samples were rinsed in ultrapure water, and leaves, roots, flowers and stems, which were harvested from three individual *C. monnieri* plants, were placed in centrifuge tubes, frozen in liquid nitrogen immediately and stored at −80 °C.

Total RNA was extracted from each tissue using RNA plant Plus Reagent (Tiangen, Beijing, China) according to the manufacturer’s instructions. The extracted RNA was checked using a NanoDrop 2000 (Thermo, CA, USA), and the concentration of the isolated RNA, 28S/18S and integrity (RIN) were estimated using an RNA Nano 6000 Assay Kit and the Agilent Bioanalyzer 2100 system (Agilent, CA, USA) (Table S1).

### Determination of osthole content

Dried *C. monnieri* samples from flowers, roots, leaves and stems were used for isolation of osthole as previously reported (Jialei et al., 2014). Dried powder (0.1 g) from each sample was mixed with ethanol (25 mL), incubated for 2 h at room temperature (25 °C) and subjected to ultrasonic extraction for 60 min (100 W, 50 kHz). The supernatant was collected, and detected using UV-spectrophotometry at 322nm (SHIMADZU Coporation, kyoto, Japan). Osthole (purity >99.5%) (Aladdin Industrial Corporation, Shanghai, China) was used to construct a standard curve of the relationship between concentration and absorbance (Fig. S1A). The yield (percentage) of osthole was calculated as follows:

\begin{eqnarray*}\text{Yield}(\text{%})= \frac{\text{Osthole content of extraction}  \left( \mathrm{g} \right) }{C.~monnieri \text{tissue powder weight}  \left( \mathrm{g} \right) } \times 100\text{%} \end{eqnarray*}

### cDNA library construction and sequencing

Total RNA was treated with RNase-free DNase I (TaKaRa, China) to remove DNA residues and then mixed with magnetic beads containing oligo (dT) to purify mRNA. After purification, the mRNA was fragmented into small pieces under elevated temperature. The cleaved RNA fragments were copied into first-strand cDNA using reverse transcriptase and random primers. Second-strand cDNA was synthesized using DNA Polymerase I and RNase H. These cDNA fragments had the addition of a single adenine at the 3′ end for subsequent ligation of adapters. The products were then purified and enriched using PCR amplification. The quality of each sample library was evaluated using an Agilent 2100 Bioanalyzer system (ABI, New York, NY, USA). Each cDNA library was sequenced using the BGISEQ-500 system at Beijing Genomics Institute (BGI) (Shenzhen, Guangdong province, China) with paired-end (PE) sequencing length of 100 bp.

### Analysis of RNA-Seq data and unigene functional annotation

To ensure the reliability of transcript assembly, clean reads were obtained by discarding low-quality reads, reads containing poly-N and adapter sequences using SOAPnuke software (version 1.5.2) (parameters: -l 15 -q 0.2 -n 0.05 − *i* − *A* 0.25). In the absence of a reference genome, clean reads were subjected to transcriptome assembly using Trinity software (version 2.5.1) applying the following parameters: –min_contig_length 150 –CPU 8 –min_kmer_cov 3 –min_glue 3 –bfly_opts ’-V 5 –edge-thr =0.1 –stderr’ (Grabherr et al., 2011). Assembled transcripts were clustered to discard redundant sequences using the TGI clustering (TGICL) tool (parameters: -l 40 -c 10 -v 25 -O‘-repeat_stringency 0.95 -minmatch 35 -minscore 35′) (Pertea et al., 2003), leading to the identification of non-redundant sequences, called unigenes. The quality of assembled transcripts was assessed using a single copy orthologous database (BUSCO) (version 1.22).

Assembled unigenes were annotated against the NCBI nucleotide sequences (NT) (https://www.ncbi.nlm.nih.gov/nucleotide), NCBI non-redundant protein sequences (NR) (update date: 23 June 2019) (NR at https://www.ncbi.nlm.nih.gov/, update date: 23 June 2019), clusters of euKaryotic Orthologous Groups (KOG) (http://www.ncbi.nlm.nih.gov/KOG), Kyoto Encyclopedia of Genes and Genomes (KEGG) (update date: 1 July 2018) (http://www.genome.jp/kegg/) (Kanehisa et al., 2019), and a manually annotated and reviewed protein sequence database (SwissProt) (update date: 25 May 2018) (http://www.uniprot.org/). Assembled unigenes were annotated against NR database using the software BLAST (version 2.2.23, *E*-value ≤ 1e−5). And the results of NR annotation were used for Gene Ontology (GO) (update date: 1 January 2019) annotation using Blast2GO (version v2.5.0) program (Conesa et al., 2005). Pfam (update date: September 2018) annotation was carried out using HMMER (version 3.0).

### Quantitative real-time PCR (qRT-PCR) analysis of key genes in coumarin biosynthesis

Total RNA from each sample was subjected to reverse transcription using SuperScript™ III First-Strand Synthesis SuperMix (Invitrogen, US). qRT-PCR analysis of each gene was performed on a QuantStudio^®^ Real-time PCR system (Life Technologies, US) using Power SYBR^®^ Green PCR Master Mix (Roche, China). The *actin* gene (CL3748.Contig7) of *C. monnieri* was used as a reference. Primers for all selected genes were designed using Premier 6.0 and are listed in Table S2. Each qRT-PCR contained 1µl of diluted cDNA, 0.5 µl of each primer (10 µM) and 10.0 µl of Power SYBR^®^ Green Master Mix. Reactions were conducted under the following conditions: denaturation at 95 °C for 1 min, followed by 40 cycles of 95 °C for 15 s and 63 °C for 25 s. Each qRT-PCR was performed using three biological replicates. The 2^−ΔΔ*Ct*^ method was used to calculate the relative expression level of genes (Livak & Schmittgen, 2001).

### Analysis of differentially expressed genes (DEGs)

After assembly, clean reads were mapped to unigenes using Bowtie2 (version 2.2.5) (Langmead & Salzberg, 2012), and then gene expression levels were calculated with RSEM (version 1.1.12) (Dewey & Li, 2011). Since we had no RNAseq bioreplicates, we used a Poisson distribution method to identify candidate genes that might be differentially expressed. Both fold change (≥2.0) and false discovery rate (≤0.001) are used as criteria to narrow down the list of differentially expressed genes between flowers and other tissues (Bullard et al., 2010; Lee et al., 2008; Li et al., 2013). qRT-PCR with bioreplicates was used to confirm differential expression of a subset of these. GO and KEGG analyses were again performed on the DEGs following the method described by Audic (Audic & Claverie, 1997).

### Identification of transcription factors

The open reading frames (ORFs) of unigenes were initially detected using getorf (version EMBOSS:6.5.7.0) (Rice, Longden & Bleasby, 2000) and further aligned to the plant transcription factor database (PlntfDB) through comparison with Pfam 23.0 using the hmmsearch method (Mistry et al., 2013). The function of unigenes was identified according to the characteristics of each transcription factor family described by PlantfDB.

### Statistical analysis

The contents of osthole and all data from qRT-PCR were presented as mean ± standard deviation. Statistics were done by GraphPad Prism 6.0. Heatmap and boxplot were performed using R software (version 3.4.4). The one-way analysis of variance (ANOVA) was used to compare the data between groups with Duncan t test. *P* value <0.05 was considered to be statistically significant.

## Results

### Osthole content determination in different tissues of *C. monnieri*

We extracted osthole from dried leaves, stems, flowers and roots of *C. monnieri*. Osthole content was highest in flowers (1.372%) and lowest in stems (0.283%) (Fig. S1B). Osthole quantification revealed significant differences among different tissues, with this active ingredient mainly distributed in flowers and secondly in leaves.

### RNA sequencing and de novo assembly

Four mRNA libraries were extracted from leaf, stem, flower and root tissue of *C. monnieri* using BGISEQ-500 sequencing technology. After eliminating low-quality reads, we obtained a total of 10.6 Gb, 10.47 Gb, 10.81 Gb and 10.38 Gb of clean reads from 83.42 Mb, 81.65 Mb, 84.27 Mb and 82.14 Mb sets of raw data from leaf, stem, flower and root tissue, respectively, with all Q30 values greater than 85% (Table S3). The clean reads were assembled using Trinity and the TGI clustering tool (TGICL). After assembly, a total of 173,938 unigenes were obtained from the four libraries with a mean length of 1,272 bp, GC content of 38.79% and N50 value of 2,121 bp (Table S4). Of these unigenes, 45.80% (79,658) and 64.13% (111,562) exceeded 1,000 bp and 500 bp in length, respectively (Fig. S2). The quality of the assembled transcripts was assessed using a single copy orthologous database (BUSCO), and 98% of the unigenes showed a perfect match (Fig. S3).

### Unigene functional annotation and expression overview

**Figure 1:**
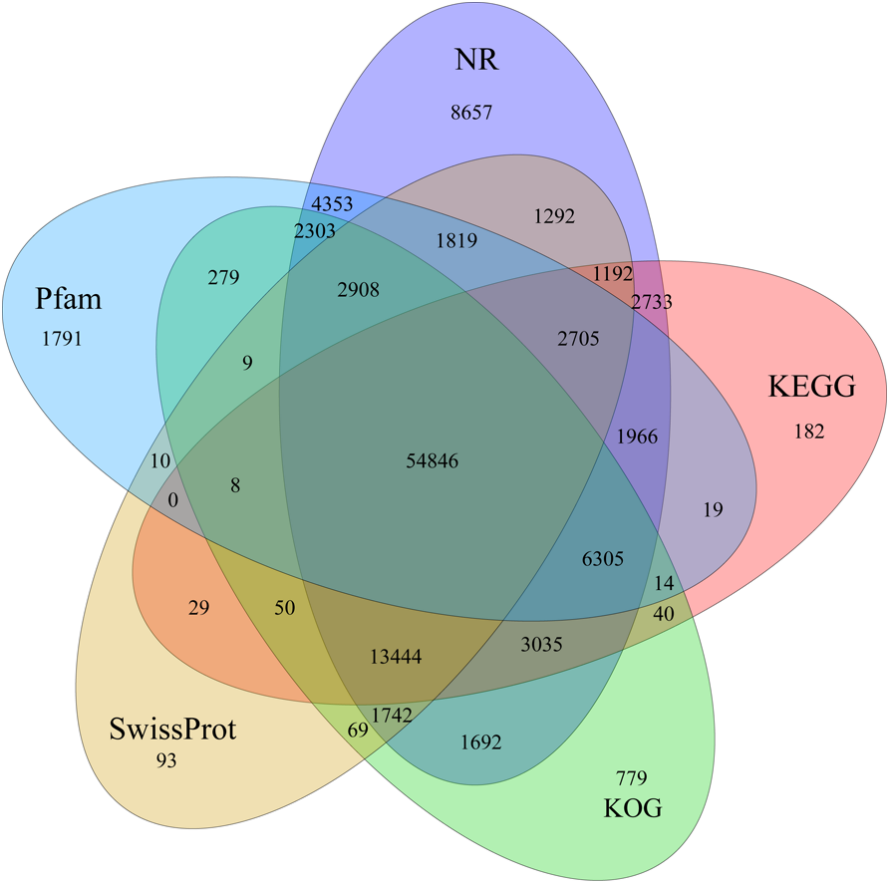
Venn diagram of annotated unigenes from different databases.

Identification of assembled unigenes was carried out using the Basic Local Alignment Search Tool (BLAST) (e <10^−5^) against seven public databases (NR, NT, Swissprot, KEGG, KOG, Pfam and GO), and a total of 110,992 (63.81%), 95,603 (54.96%), 80,216 (46.12%), 86,568 (49.77%), 87,523 (50.32%), 79,335 (45.61%) and 56,429 (32.44%) unigenes were aligned, respectively (Table 1). We annotated 56,429 (32.44%) unigenes using GO terms classified into 58 subcategories within three standard categories (molecular functions, biological processes and cellular components) (Fig. S4). We focused on the categories of molecular functions and biological processes. “Catalytic activity” and “binding” were the most enriched terms among the molecular functions, while “cell process” and “metabolic process” were the top subcategories of biological processes. According to Venn diagram analysis, 54,846 (31.53%) unigenes were co-annotated in five databases (Fig. 1). Additionally, 110,992 unigenes were annotated in the NR database and 55.48% of these annotated unigenes were mapped to *Daucus carota* subsp. *sativus* (82.2%) and others (17.8%) (Fig. S5).

**Table 1:** Summary of *C. monnieri* unigenes annotated via seven public databases.

**Database**	**Number annotated**	**Annotated unigene ratio (%)**
NR	110,992	63.81
NT	95,603	54.96
Swissprot	80,216	46.12
KOG	87,523	50.32
KEGG	86,568	49.77
Pfam	79,335	45.61
GO	56,429	32.44
Overall	119,177	68.52

**Figure 2:**
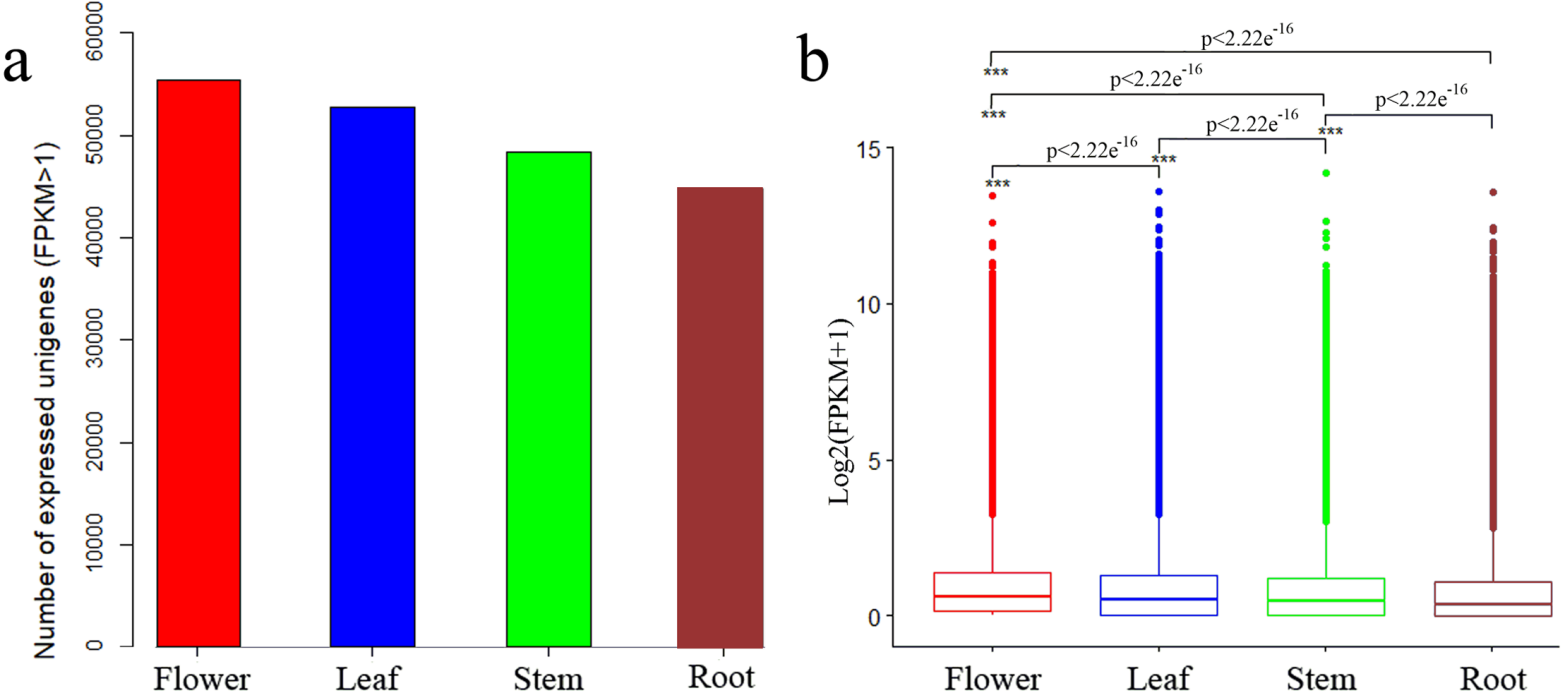
Expression profiles in *C. monnieri* flower, leaf, stem and root tissues. (A) Distribution of number of expressed unigenes (FPKM > 1) in the four tissues. (B) Boxplot of unigenes expressed among the four tissues. The x-axis represents the four tissues, and the *y*-axis represents log2 (FPKM + 1) values. *p* < 2.22*e*^−16^ indicates a significant difference among the four tissues. Significant difference was detected using the Kruskal-Wallis nonparametric test.

We determined expression values of transcripts with fragments per kilobase of transcript per million mapped reads (FPKM) >1 for each tissue and found 55,421, 52,787, 48,287 and 44,717 expressed unigenes in flower, leaf, stem and root tissues, respectively (Fig. 2A). The overall expression level of unigenes in flowers was higher than that in leaves, stems or roots (Fig. 2B).

### Identification of genes involved in coumarin biosynthesis

**Figure 3:**
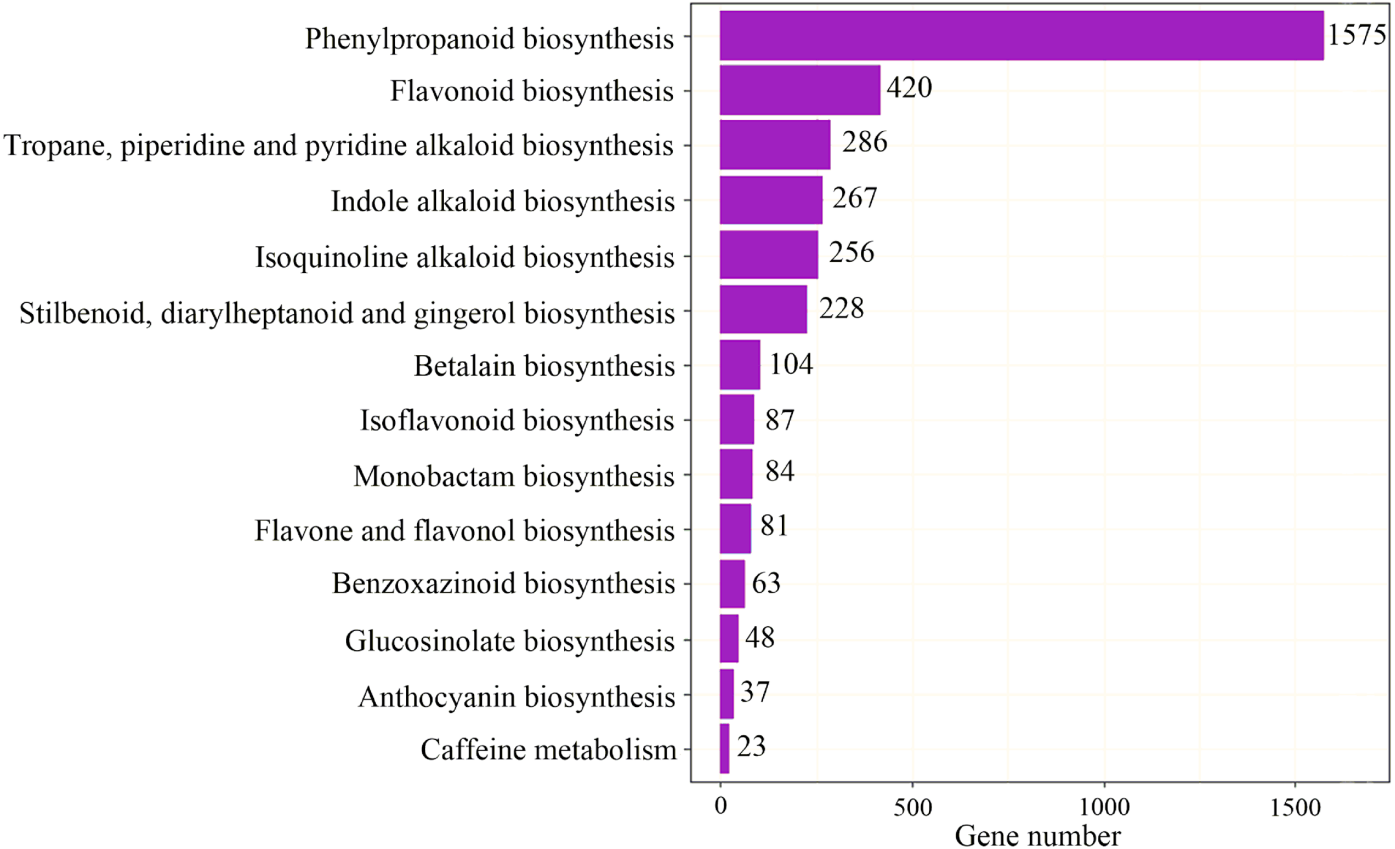
Pathway classification for biosynthesis of other secondary metabolites.

**Figure 4:**
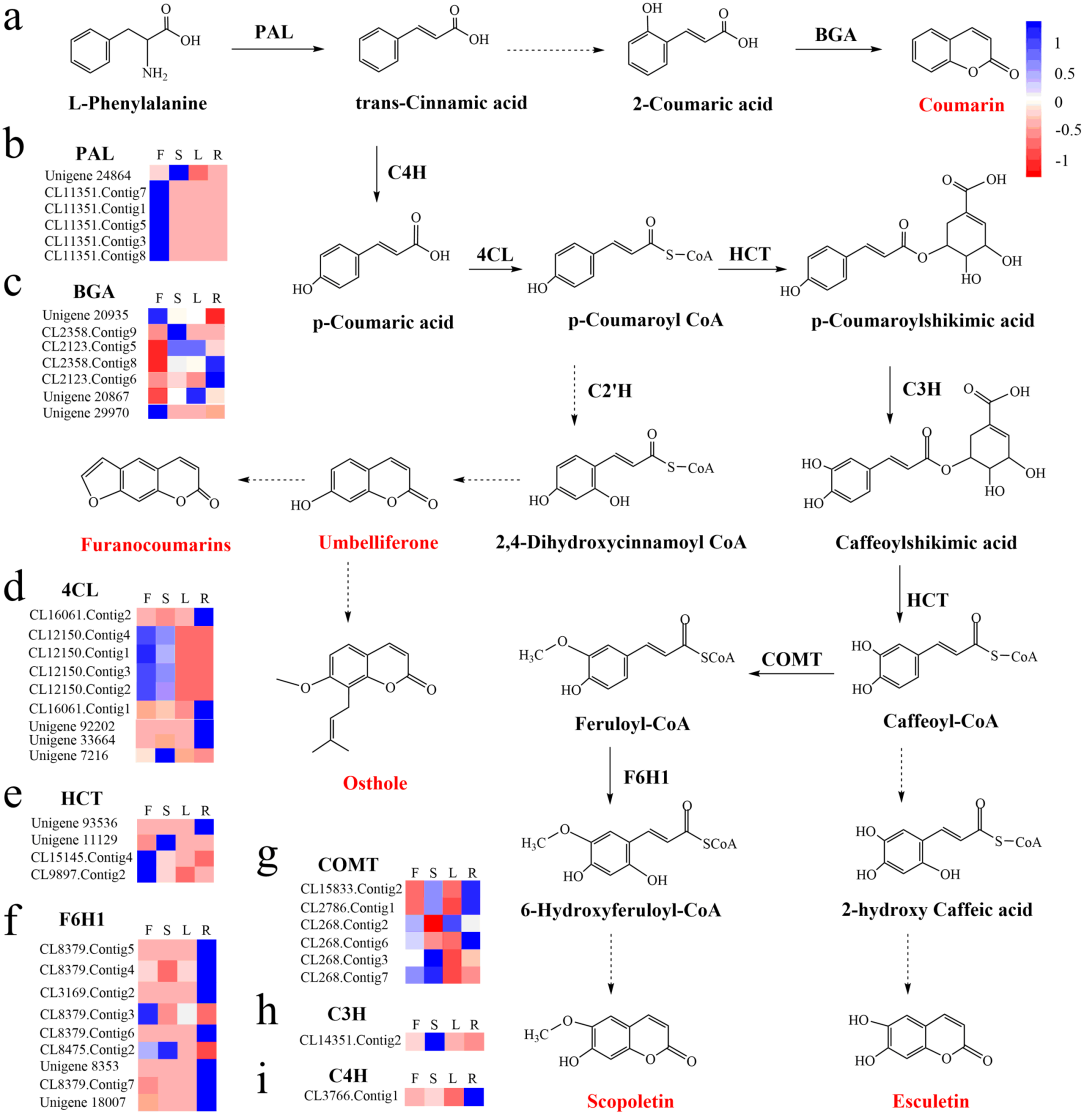
Proposed pathway for coumarin biosynthesis in *C. monnieri* (A) and the expression levels of unigenes encoding enzymes involved in each step are shown (B–I). Columns L, R, S and F represent leaf, root, stem and flower tissues, respectively. Blue and red represent high and low expression level, respectively.

To identify the most significant biological processes in the leaf, stem, flower and root tissues of *C. monnieri*, 86,568 (49.77%) unigenes were annotated and assigned to 135 KEGG pathways (19 subcategories) (Fig. S6, Table S5). Fourteen pathways were associated with the biosynthesis of other secondary metabolites, of which phenylpropanoid biosynthesis pathway was most enriched with annotated genes (Fig. 3). Phenylpropanoids comprise a large group of natural products including coumarins, flavonoids and lignins. In our transcriptome data, 1,575 unigenes were assigned to the phenylpropanoid biosynthesis (ko00940) pathway based on KEGG pathway classification. Of these, 853 unigenes encode enzymes participating in the coumarin biosynthesis pathway: phenylalanine ammonia-lyase (PAL, 14 unigenes), *β*-glucosidase (BGA, 507 unigenes), cinnamate 4-hydroxylase (C4H, 2 unigenes), 4-coumarate–CoA ligase (4CL, 49 unigenes), shikimate hydroxycinnamoyl transferase (HCT, 70 unigenes), 5-O-(4-coumaroyl)-D-quinate 3′-monooxygenase (C3′H, 95 unigenes), caffeoyl-CoA O-methyltransferase (COMT, 33 unigenes) and feruloyl-CoA ortho-hydroxylase (F6′H1, 83 unigenes) (Table 2). These data enabled us to outline coumarin biosynthesis based on genes encoding enzymes with FPKM >1 (Fig. 4).

**Table 2:** Number of unigenes encoding enzymes involved in coumarin biosynthesis in *C. monnieri*.

**Enzyme name**	**EC number**	**Number of unigenes**	**No. in flower**	**No. in leaves**	**No. in roots**	**No. in stems**
Phenylalanine ammonia-lyase, PAL	4.3.1.24	14	14	6	5	5
*β*-Glucosidase, BGA	3.2.1.21	507	434	375	327	347
Cinnamate-4-hydroxylase, C4H	1.14.14.91	2	2	2	2	2
4-Coumarate-CoA ligase, 4CL	6.2.1.12	49	36	24	35	30
Shikimate hydroxycinnamoyl transferase, HCT	2.3.1.133	70	61	45	45	47
5-O-(4-Coumaroyl)-D-quinate 3′-monooxygenase, C3′H	1.14.14.96	95	94	92	84	93
Caffeoyl-CoA O-methyltransferase, COMT	2.1.1.104	33	27	26	28	28
Feruloyl-CoA ortho-hydroxylase, F6′H1	1.14.11.-	83	71	73	66	71

### Expression analysis of key enzyme genes

We selected six unigenes encoding five key enzymes and used quantitative real-time PCR (qRT-PCR) to determine their relative expression levels in the leaf, stem, flower and root tissues of *C. monnieri* (Fig. S7). Relative expression of the CL3766.Contig1 (*C4H*), CL16061.Contig1 (*4CL*) and CL8379.Contig6 (*F6H1*) genes was highest in root tissue, whereas relative expression of the CL11351.Contig3 (*PAL*) and CL2358.Contig9 (*BGA*) genes was highest in flower tissue. Unigene 20867 (*BGA*) had a high expression level in leaf tissue. The expression levels determined by qRT-PCR were consistent with the FPKM values.

### Identification of DEGs

**Figure 5:**
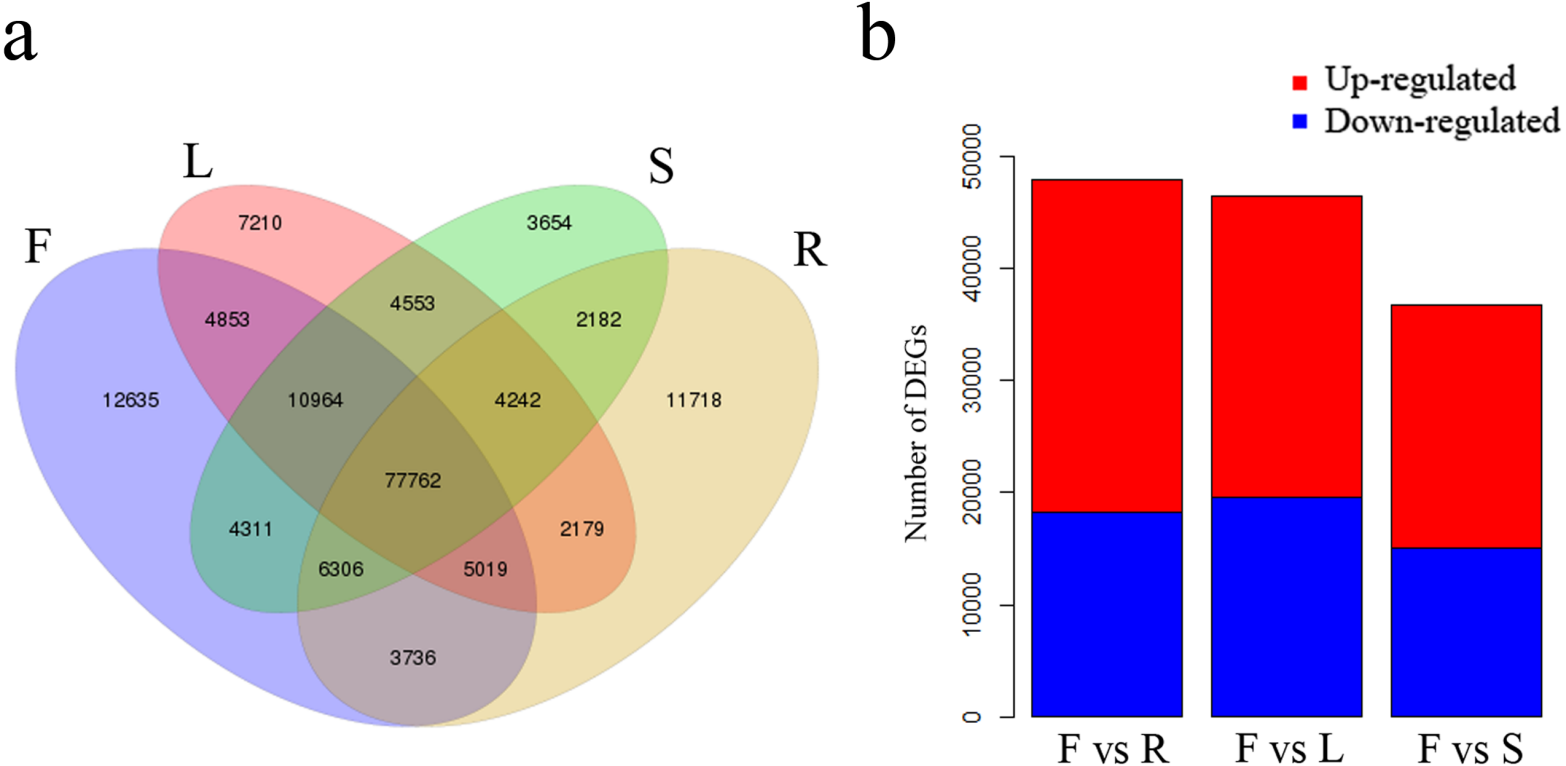
Unigenes expressed in flowers, leaves, stems and roots of *C. monnieri*. (A) Venn diagram of unigenes expressed in the four tissues. (B) Number of differentially expressed genes (DEGs) in the four *C. monnieri* tissues. DEGs with high or low expression level in flowers compared with the other three types of tissues are defined as “up-regulated” or “down-regulated,” respectively. The columns L, R, S and F represent leaf, root, stem and flower tissues, respectively.

We identified 12,635 unigenes uniquely expressed in flowers and 77,762 shared unigenes exhibiting expression in all four tissues (Fig. 5A). Total DEGs were identified among the four tissues using gene FPKM values. A comparison between flowers and leaves revealed a total of 46,460 DEGs, of which 26,944 were up-regulated and 19,516 were down-regulated in flowers (Fig. 5B). A further comparison of flowers with roots revealed 47,901 DEGs, of which 29,754 were up-regulated and 18,147 were down-regulated in flowers (Fig. 5B). Comparison among flowers and stems revealed 36,666 DEGs, of which 21,683 were up-regulated and 14,983 were down-regulated in flowers (Fig. 5B).

All DEGs were annotated in the KEGG database to further describe and evaluate their biological functions. This analysis revealed 32,060 DEGs in flower versus leaf associated with 137 pathways; these DEGs were mainly enriched in the “plant hormone signal transduction,” “plant-pathogen interaction,” “circadian rhythm - plant” and “photosynthesis - antenna proteins” pathways (Table S6). In flower versus root, 34,786 DEGs were annotated to 137 pathways and primarily enriched in “biosynthesis of secondary metabolites,” “photosynthesis,” “photosynthesis - antenna proteins” and “phenylpropanoid biosynthesis” pathways (Table S7). In flower versus stem, 27,513 DEGs were annotated to 137 pathways and primarily enriched in “plant hormone signal transduction,” “indole alkaloid biosynthesis,” “glucosinolate biosynthesis” and “phenylpropanoid biosynthesis” pathways (Table S8).

A total of 18,881 up-regulated DEGs showed flower-specific expression with |log2 (fold changes) | >2. We inferred the nature of these DEGs via GOSlim functional analysis. Sequence homology revealed that each of the 18,881 flower-specific DEGs mapped to at least one ontology term, including 2,814 for biological processes, 3,232 for cellular components and 4,508 for molecular function; many genes were enriched in “cellular process” and “catalytic activity” subcategories within the biological processes and molecular function categories, respectively (Fig. S8).

**Figure 6:**
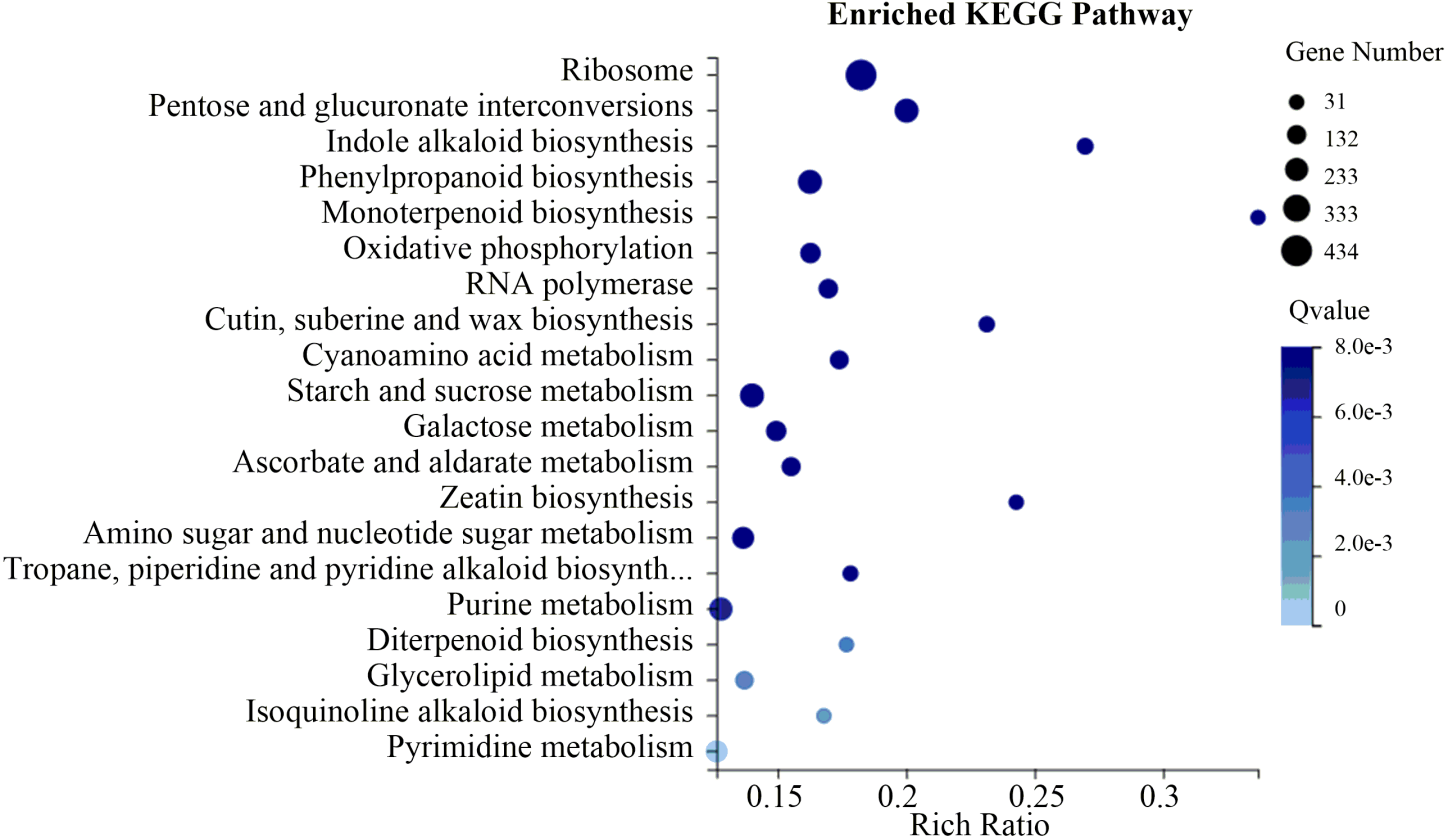
Flower-specific DEGs enriched in the KEGG pathway.

To evaluate the biological functions of these DEGs, the 18,881 flower-specific DEGs were annotated in the KEGG database. KEGG enrichment analysis showed that these DEGs were significantly enriched in the “ribosome,” “starch and sucrose metabolism,” “pentose and glucuronate interconversions” and “phenylpropanoid biosynthesis” categories (Fig. 6). Additionally, we identified 129 up-regulated flower-specific DEGs with —log2 (fold change)—>2 related to coumarin biosynthesis, including BGA (99 DEGs), 4CL (2 DEGs), HCT (13 DEGs), COMT (7 DEGs) and F6′H1 (8 DEGs) (Table 3, Table S9).

**Table 3:** Number of up-regulated unigenes in coumarin biosynthesis.

**Abbreviation**	**EC number**	**Number of up-regulated unigenes**			
		**Flower vs Leaf**	**Flower vs Stem**	**Flower vs Root**	**Flower–specific expressed**
PAL	4.3.1.24	6	9	5	0
BGA	3.2.1.21	301	312	358	99
C4H	1.14.14.91	2	1	1	0
4CL	6.2.1.12	26	23	26	2
HCT	2.3.1.133	42	48	41	13
C3H	1.14.14.96	12	27	89	0
COMT	2.1.1.104	18	14	18	7
F6′H1	1.14.11.−	40	47	38	8

### Identification of transcription factors (TFs)

Transcription factors (TFs) play an important role in regulating secondary metabolites by modulating the expression of genes related to biosynthetic pathways. We identified 3,799 unigenes encoding putative TFs in the *C. monnieri* transcriptome database, including 1,169, 1,032 and 920 unigenes up-regulated in flowers compared with leaves, stems and roots, respectively (Table 4). The major types of TF identified belonged to the MYB (v-myb avian myeloblastosis viral oncogene homolog) (564 unigenes), AP2-EREBP (APETALA2 and ethylene-responsive element binding proteins) (285 unigenes), bHLH (Basic helix-loop-helix) (274 unigenes), NAC (222 unigenes), C3H (Cys3His zinc finger) (193 unigenes), WRKY (191 unigenes), and C2H2 (C2H2-type transcription factor) (173 unigenes) TF families. Moreover, a total of 15 unigenes encoding 3 TFs (MYB TF, GRAS TF and HAT TF) participated in the biosynthesis of phenylpropanoid (Table S10). Of these, 7 unigenes encoding MYB TF were up-regulated with flower-specific expression; these unigenes are shown in red in Table S10.

**Table 4:** Classification and number of TF families identified in the DEGs database of *C. monnieri*.

**TF family**	**Number of unigenes**	**Number of up-regulated unigenes**		
		**Flower vs Leaf**	**Flower vs Stem**	**Flower vs Root**
MYB	564	166	145	122
AP2-EREBP	285	78	59	57
bHLH	274	86	79	71
NAC	222	59	30	41
C3H	193	65	53	39
WRKY	191	36	16	21
C2H2	173	30	39	26
ABI3VP1	151	43	47	46
MADS	143	48	56	56
G2-like	116	35	39	27
ARF	103	44	29	27
mTERF	93	30	50	35
C2C2-GATA	87	18	19	22
GRAS	87	18	14	16
Trihelix	81	37	31	23
SBP	72	34	30	26
LIM	69	22	21	19
Tify	68	3	3	8
FAR1	59	17	15	12
C2C2-Dof	53	23	12	10
Alfin-like	51	15	10	12
TCP	51	26	26	20
Other	538	157	139	127
Total number	3,949	1,169	1,032	920

## Discussion

Coumarins are the most important bioactive components of *C. monnieri*. Their structures and physicochemical properties have been well studied (Yang et al., 2015). However, their biosynthetic and metabolic pathways are poorly investigated, mainly owing to the absence of genomic or transcriptomic resources. Thus, understanding the biosynthesis, metabolism and regulation of coumarins will facilitate the further investigation of *C. monnieri*. We used four different types of tissues for RNA-Seq analysis using the BGI-500 platform and generated 173,938 unigene sequences with an N50 of 2,121 bp, GC content of 38.79% and an average length of 1,272 bp. In addition, 119,177 (68.52%) genes were mapped in at least one public database, while 54,761 (31.48%) genes remained unannotated because of the lack of available genomic information.

The KEGG database is a useful tool for determining candidate genes in biological processes (Liu et al., 2017; Shi et al., 2019; Wang et al., 2018). We identified numerous unigenes participating in coumarin biosynthesis through KEGG annotations. Expression levels of unigenes encoding enzymes responsible for coumarin biosynthesis were determined based on FPKM values. Unigenes encoding PAL (CL11351.Contig1,3,5,7,8), HCT (CL15145.Contig4, CL9897.Contig2) and 4CL (CL12150.Contig1,2,3,4) were highly differentially expressed in flower compared to other tissues. By contrast, those encoding BGA (CL2123.Contig6, CL2358.Contig9), C4H (CL3766.Contig1), 4CL (CL16061.Contig1,2, Unigene 33664, Unigene 92202) and F6H1 (CL8379.Contig4,5,6,7, CL3169.Contig2, Unigene 8533, Unigene 18007) were highly differentially expressed in root compared to other tissues. The mRNA expression levels of CL11351.Contig3 (*PAL*), CL2358.Contig9 (*BGA*), Unigene 20867 (*BGA*), CL3766.Contig1 (C4H), CL16061.Contig1 (4CL) and CL8379.Contig6 (F6H1) examined using qRT-PCR were consistent with the FPKM values determined by RNA-Seq. Coumarins are a major group of natural plant products derived from the phenylpropanoid pathway (Hawryl, Soczewinski & Dzido, 2000; Lin et al., 2013), and PAL, BGA, C4H, 4CL and F6′H1 are key enzymes in coumarin biosynthesis (Poulton, Mcree & Conn, 1980; Yang et al., 2015). Studies have shown that the expression level of *PAL* is increased under methyl jasmonate, UV irradiation and cold treatment, resulting in the improvement of coumarin content (Sui et al., 2019). Overexpression of *AgC4H* or *AgPAL* genes promotes the production of decursinol angelate (pyranocoumarins) in *Angelica gigas* (Park, Park & Park, 2011). These key enzymes play important roles in regulating coumarin metabolism, and the expression levels of their genes can affect the accumulation of coumarins.

KEGG enrichment analysis showed that 129 up-regulated DEGs with flower-specific expression encoding BGA (99 DEGs), 4CL (2 DEGs), HCT (13 DEGs), COMT (7 DEGs) and F6′H1 (8 DEGs) were related to coumarin biosynthesis (Table 3). These key enzymes catalyzed the production of different structural coumarins, such as osthole, scopoletin, umbelliferone and esculetin (Fig. 4). The high expression levels of these key enzyme genes were consistent with the high accumulation of coumarins in *C. monnieri* flowers, as revealed by UV spectrophotometry. Thus, these genes may play critical roles in regulating the production and accumulation of coumarins in *C. monnieri* flowers.

Transcription factors play an important role in the growth and development of higher plants as well as their responses to the environment (Chen, 2013). We assigned 3,799 unigenes to the MYB, AP2-EREBP, bHLH, NAC, C3H, WRKY and C2H2 families. MYB TFs comprise one of the largest TF families and are important for controlling biochemical processes such as responses to biotic and abiotic stresses, metabolism and signal transduction of hormones (Qing et al., 2009). In the secondary metabolism of plants, MYB TFs mainly participate in the phenylpropanoid metabolic pathway; coumarins are one of the important plant secondary metabolites belonging to the phenylpropanoids (Borevitz et al., 2000; Chezem et al., 2017; Niu, Jiang & Xu, 2016). Overexpression of genes encoding MYB TFs in *Arabidopsis* results in increased biosynthesis and accumulation of scopoletin, which belongs to the coumarins (Chezem et al., 2017). In this study, we identified 564 unigenes encoding MYB TFs, including 166 TFs up-regulated in flower versus leaf, 145 TFs up-regulated in flower versus stem and 122 TFs up-regulated in flower versus root (Table 4). Among these unigenes, 15 encoding MYB TF were related to phenylpropanoid biosynthesis, and coumarins are derived from the phenylpropanoid pathway (Bourgaud et al., 2006). These unigenes might be crucial for regulating yields of coumarin in *C. monnieri*.

## Conclusions

We performed comprehensive transcriptome analysis of flower, leaf, stem and root tissues of *C. monnieri*. Numerous genes involved in coumarin biosynthesis were identified by RNA sequencing. Validation of several genes by qRT-PCR produced results consistent with RNA-Seq data. Our results increase the understanding of relevant metabolic pathways in *C. monnieri* and provide effective gene expression profile information for *C. monnieri*.

##  Supplemental Information

10.7717/peerj.10157/supp-1 Figure S1 Determination of the osthole content in different tissues of *C. monnieri* (A) Standard curve of osthole at 322 nm. (B) Osthole content from flowers, leaves, roots and stems of *C. monnieri*. Significant difference at a level of 0.01 by one-way ANOVA using a Duncan *t* test.

10.7717/peerj.10157/supp-2 Figure S2 Length distribution of all unigenes for *C. monnieri* transcriptome assembly

10.7717/peerj.10157/supp-3 Figure S3 The results of assembled transcripts assessed using BUSCO

10.7717/peerj.10157/supp-4 Figure S4 GO functional annotation of *C. monnieri* transcriptome

10.7717/peerj.10157/supp-5 Figure S5 Species distribution of annotations from the NR database for *C. monnieri* unigenes

10.7717/peerj.10157/supp-6 Figure S6 KEGG functional classifications of the annotated unigenes in *C. monnieri*

10.7717/peerj.10157/supp-7 Figure S7 Phenylpropanoid biosynthesis based on the KEGG database (Kanehisa et al., 2019) Red boxes represent enzymes annotated in the KEGG database. Photo credit: Kanehisa Laboratories.

10.7717/peerj.10157/supp-8 Figure S8 Expression analysis of six unigenes encoding five enzymes involved in coumarin biosynthesis in *C. monnieri* Relative expression of (A) CL11351.Contig3 (PAL), (B) CL2358.Contig9 (BGA), (C) Unigene 20867 (BGA), (D) CL3766.Contig1 (C4H), (E) CL16061.Contig1 (4CL) and (F) CL8379.Contig6 (F6H1) was analyzed by qRT-PCR using the *actin gene* (CL3748.Contig7) as a reference gene for normalization. FPKM values of these genes are shown as red lines, qRT-PCR results of these genes are shown as blue bars.

10.7717/peerj.10157/supp-9 Figure S9 GOSlim analysis of up-regulated flower-specific genes

10.7717/peerj.10157/supp-10 Supplemental Information 10 Raw Data for Fig. 1

10.7717/peerj.10157/supp-11 Supplemental Information 11 Raw Data for Fig. 2

10.7717/peerj.10157/supp-12 Supplemental Information 12 Raw Data for Fig. 3

10.7717/peerj.10157/supp-13 Supplemental Information 13 Raw Data for Fig. 5A

10.7717/peerj.10157/supp-14 Supplemental Information 14 Raw Data for Fig. 5B - F vs L

10.7717/peerj.10157/supp-15 Supplemental Information 15 Raw Data for Fig. 5B - F vs R

10.7717/peerj.10157/supp-16 Supplemental Information 16 Raw Data for Fig. 5B - F vs S

10.7717/peerj.10157/supp-17 Supplemental Information 17 Raw Data for Fig. 6

10.7717/peerj.10157/supp-18 Supplemental Information S1 Supplementary materials

## Additional Information and Declarations

### Field Study Permissions

The following information was supplied relating to field study approvals (i.e., approving body and any reference numbers): Whole *C. monnieri* plants were collected from Tongcheng city in Anhui Province, China in May 2018, with verbal permission of the Manager Zhongquan Fang (Anhui Bowen agriculture and Forestry Development Co., Ltd), and authenticated by Professor Dequn Wang (Anhui University of Chinese Medicine).

### Data Availability

The following information was supplied regarding data availability: Data are available at NCBI Sequence Read Archive (SRA): SRP217313, Message Body PRJNA554518. Assembled unigenes are available at Figshare: Wu, jiawen (2020): All-Unigene-Sequence.fa. figshare. Dataset. https://doi.org/10.6084/m9.figshare.11600601.v1. Gene information is also available at Figshare: Wu, jiawen (2020): Information of the genes.xls. figshare. Dataset. https://doi.org/10.6084/m9.figshare.12764177.v1.
