# Supplementary figures and images for: De novo transcriptome analysis of Cnidium monnieri (L.) Cuss and detection of genes related to coumarin biosynthesis
Source: PeerJ. 2020 Nov 6;8:e10157. doi: 10.7717/peerj.10157 (PMC7651471; doi:10.7717/peerj.10157)

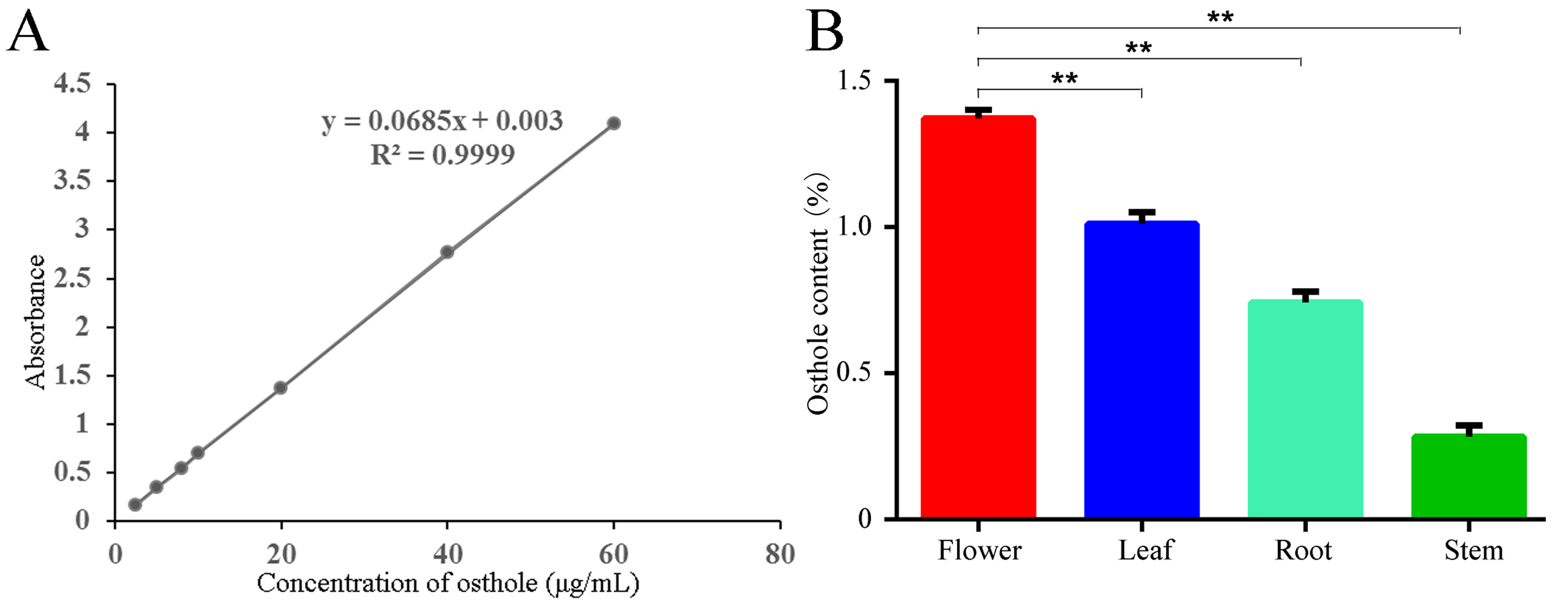

Supplement: Figure S1 — (A) Standard curve of osthole at 322 nm. (B) Osthole content from flowers, leaves, roots and stems of C. monnieri. Significant difference at a level of 0.01 by one-way ANOVA using a Duncan t test. [file peerj-08-10157-s001.png]

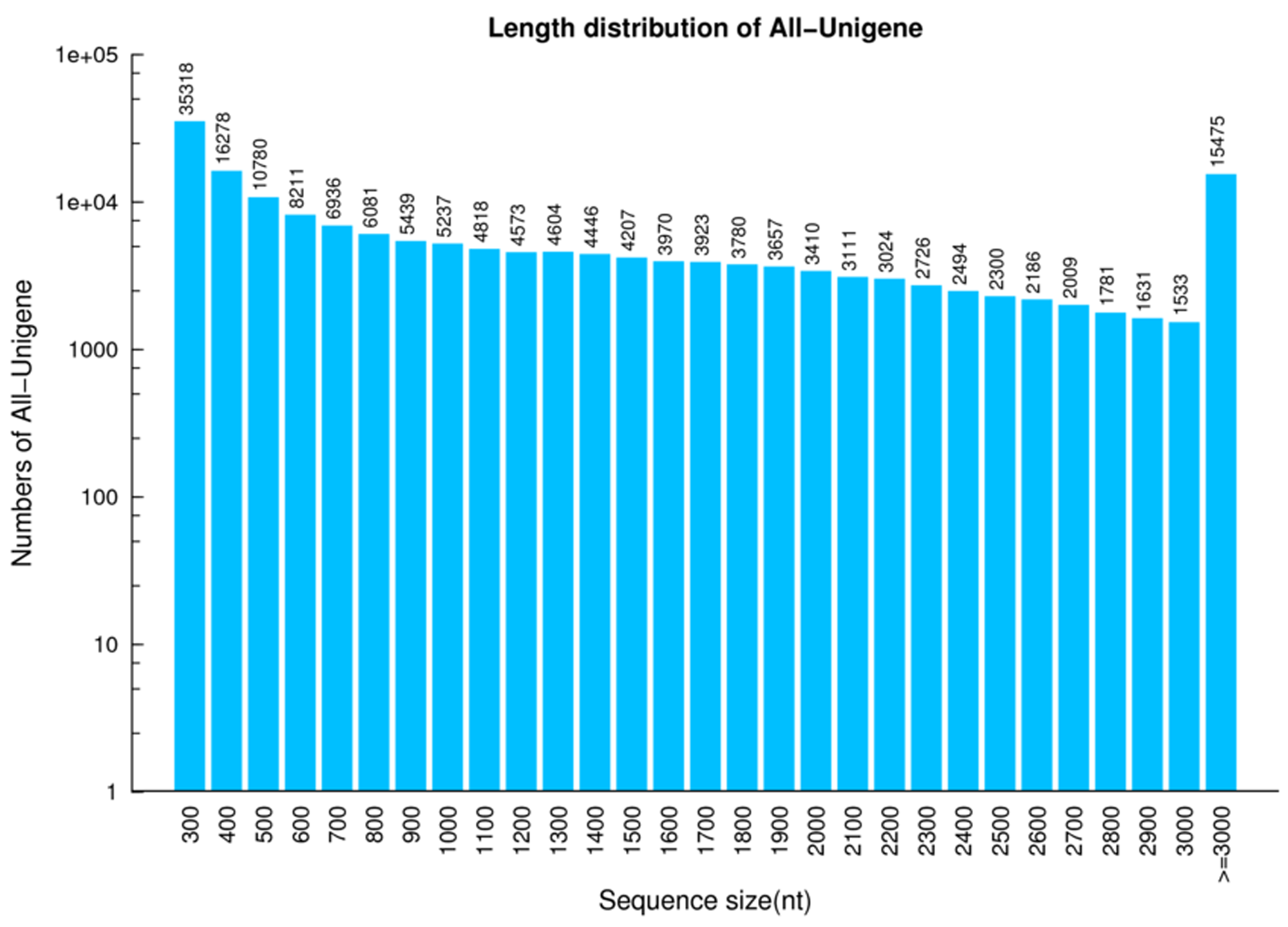

Supplement: Figure S2 [file peerj-08-10157-s002.png]

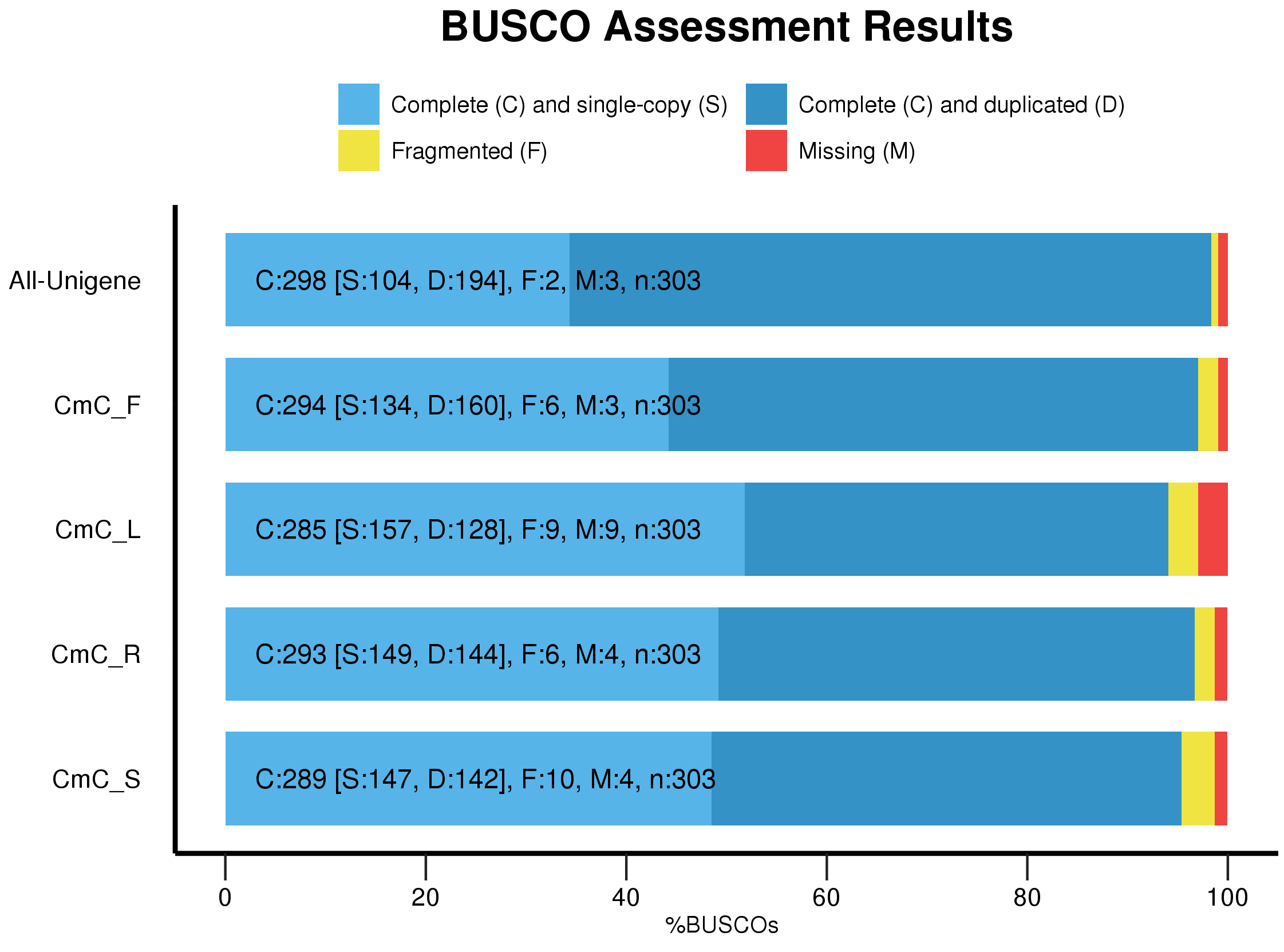

Supplement: Figure S3 [file peerj-08-10157-s003.png]

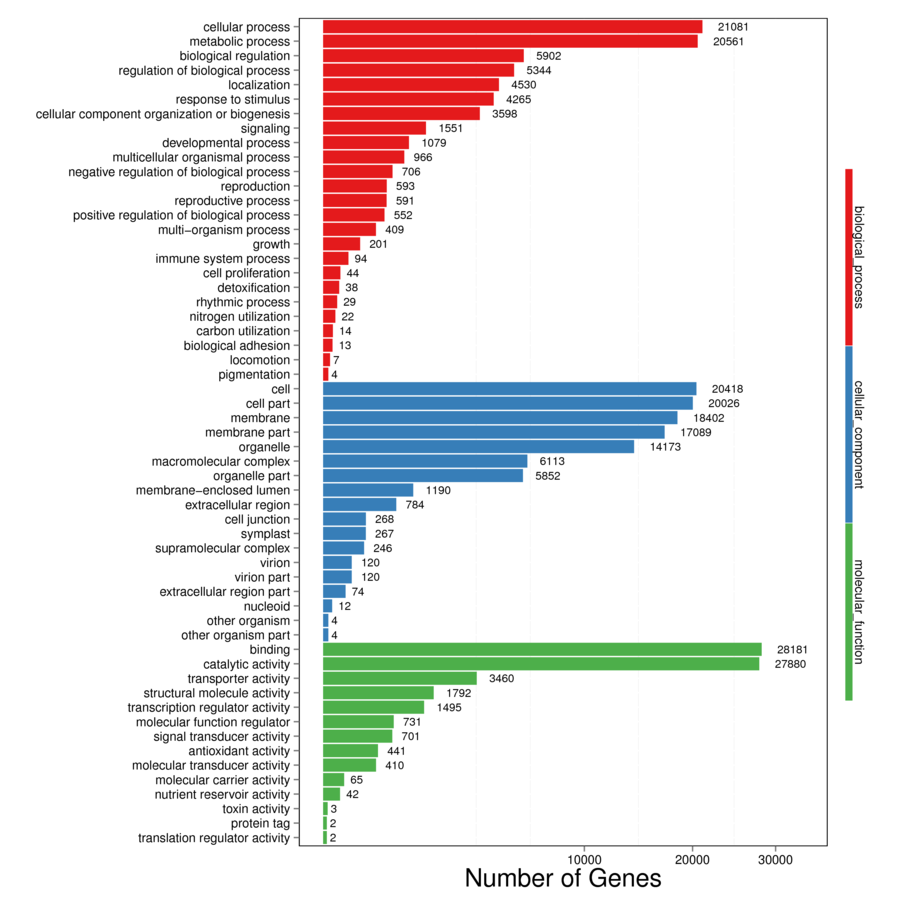

Supplement: Figure S4 [file peerj-08-10157-s004.png]

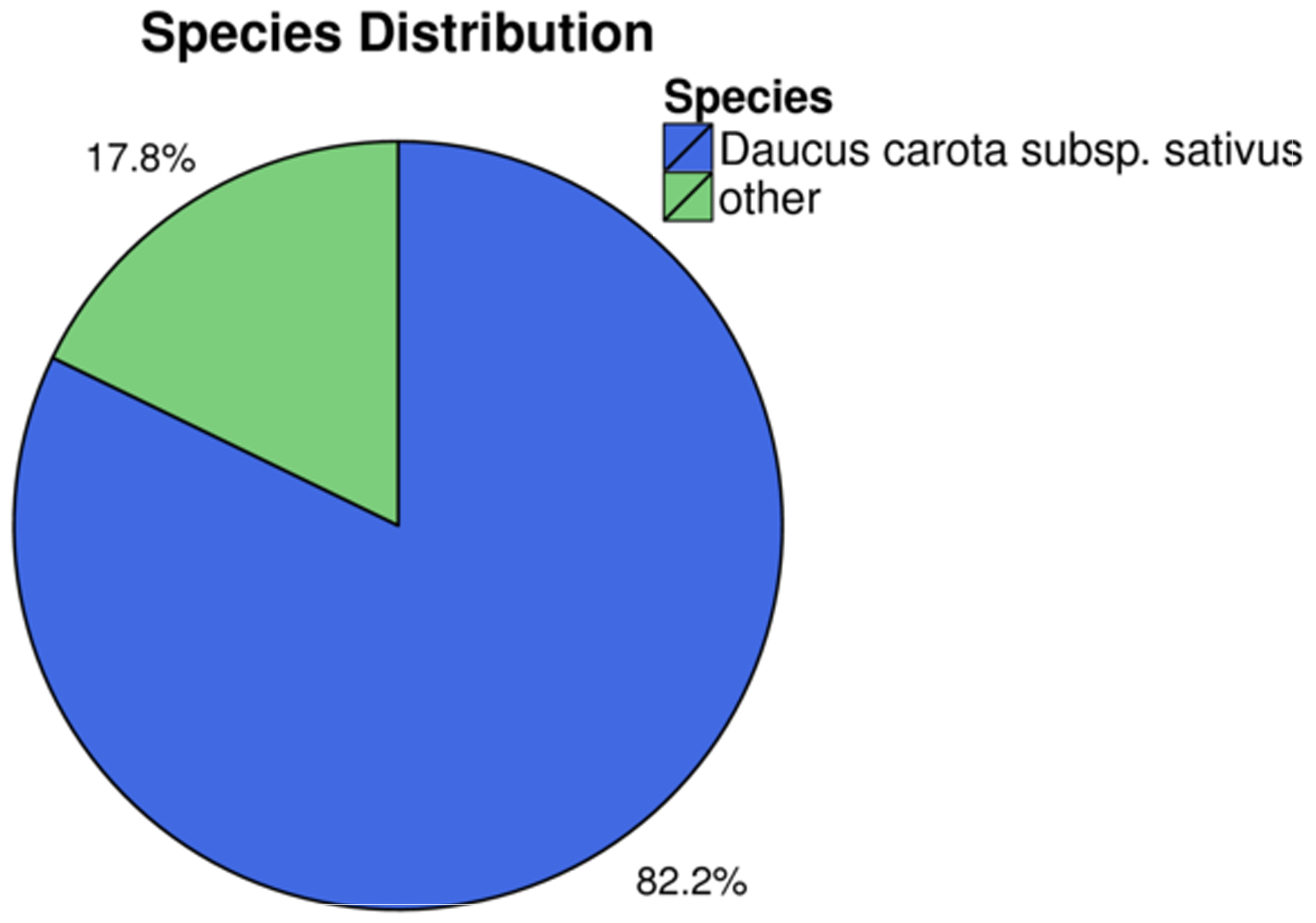

Supplement: Figure S5 [file peerj-08-10157-s005.png]

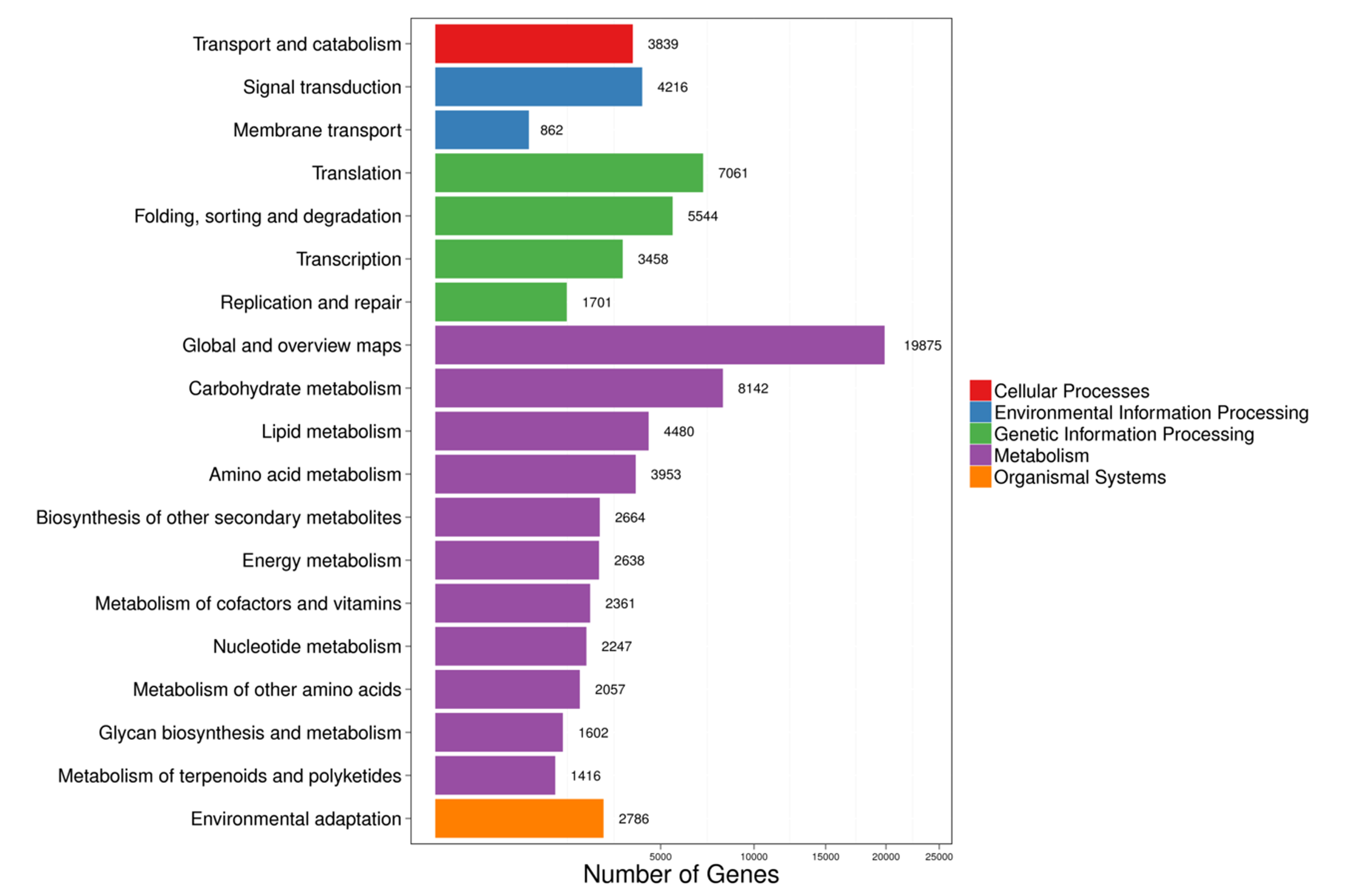

Supplement: Figure S6 [file peerj-08-10157-s006.png]

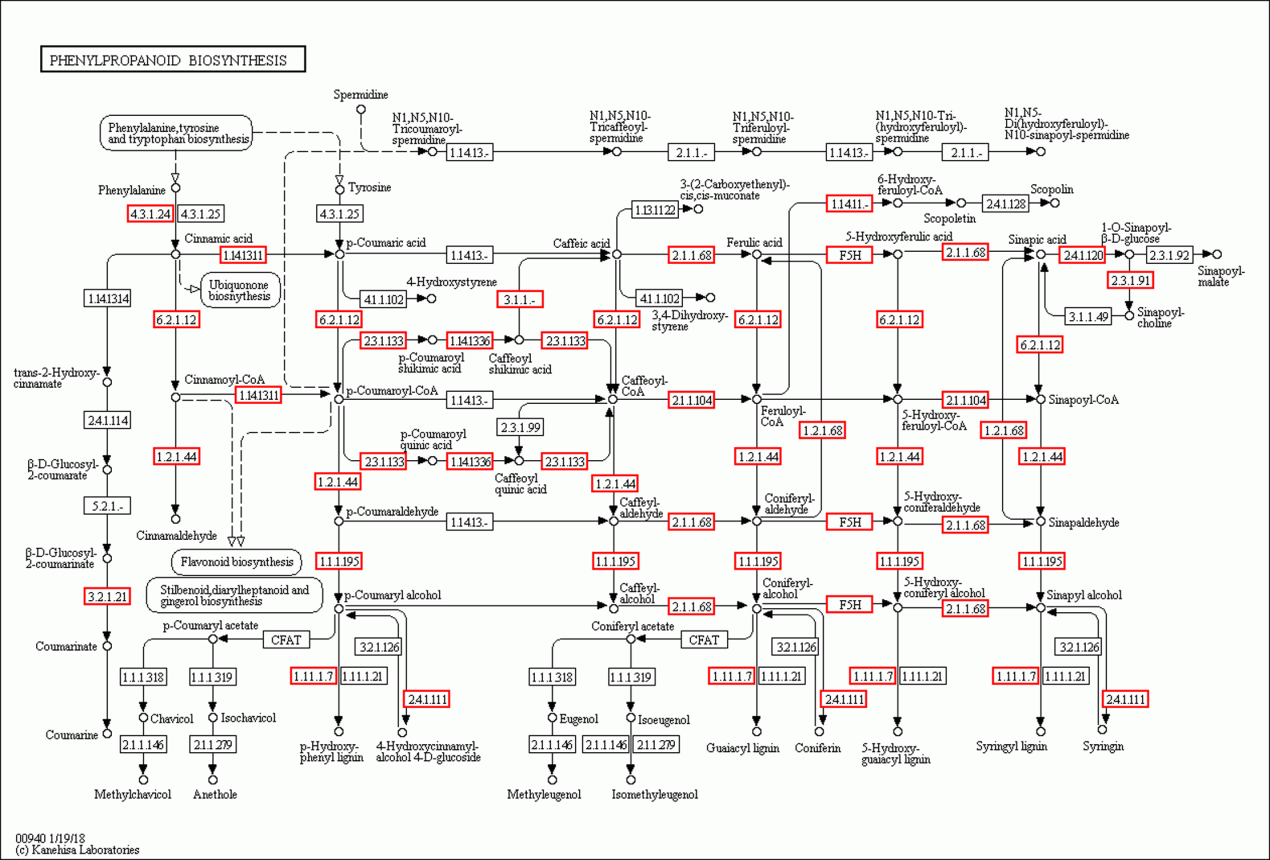

Supplement: Figure S7 — Red boxes represent enzymes annotated in the KEGG database. Photo credit: Kanehisa Laboratories. [file peerj-08-10157-s007.png]

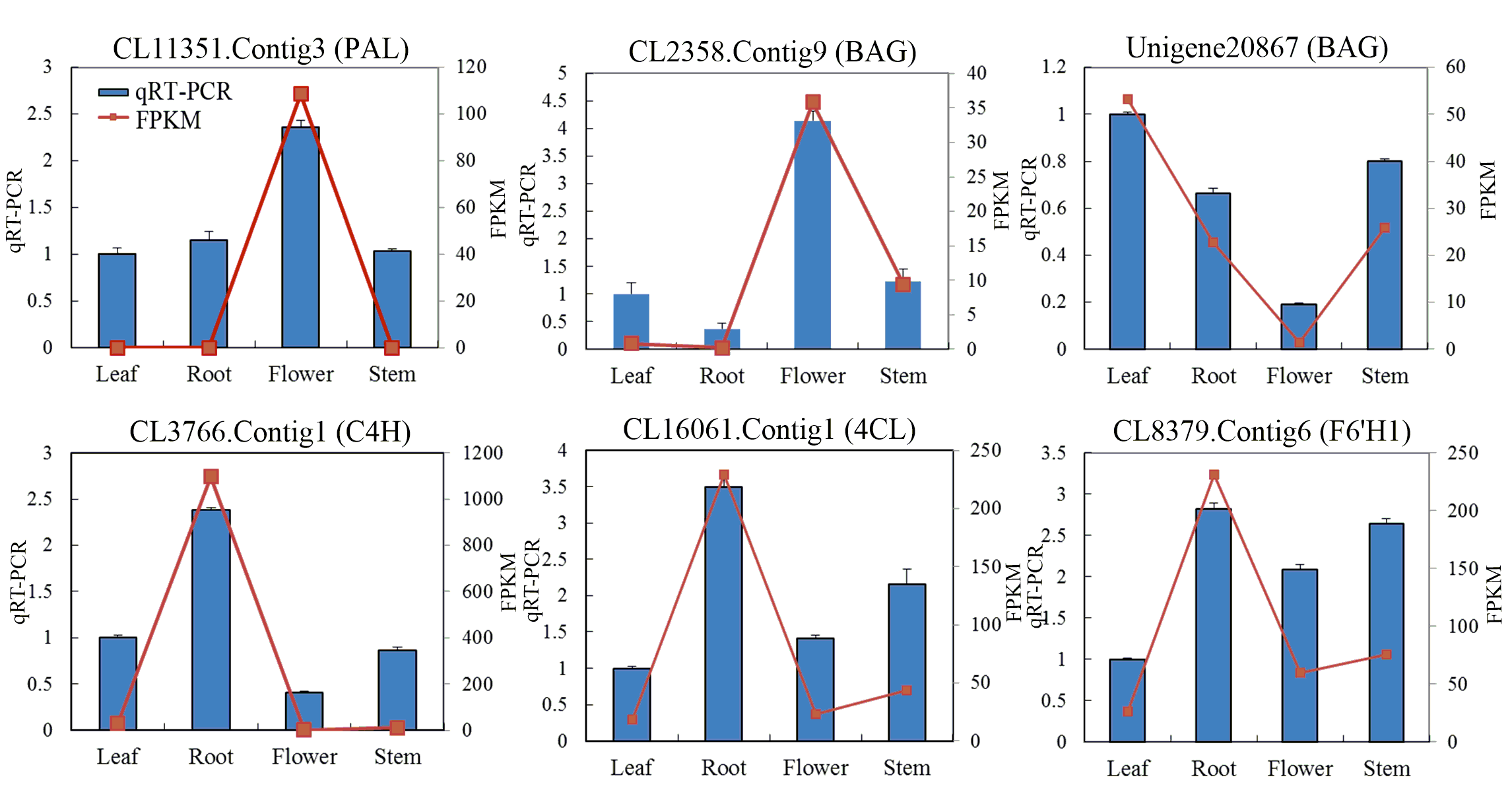

Supplement: Figure S8 — Relative expression of (A) CL11351.Contig3 (PAL), (B) CL2358.Contig9 (BGA), (C) Unigene 20867 (BGA), (D) CL3766.Contig1 (C4H), (E) CL16061.Contig1 (4CL) and (F) CL8379.Contig6 (F6H1) was analyzed by qRT-PCR using the actin gene (CL3748.Contig7) as a reference gene for normalization. FPKM values of these genes are shown as red lines, qRT-PCR results of these genes are shown as blue bars. [file peerj-08-10157-s008.png]

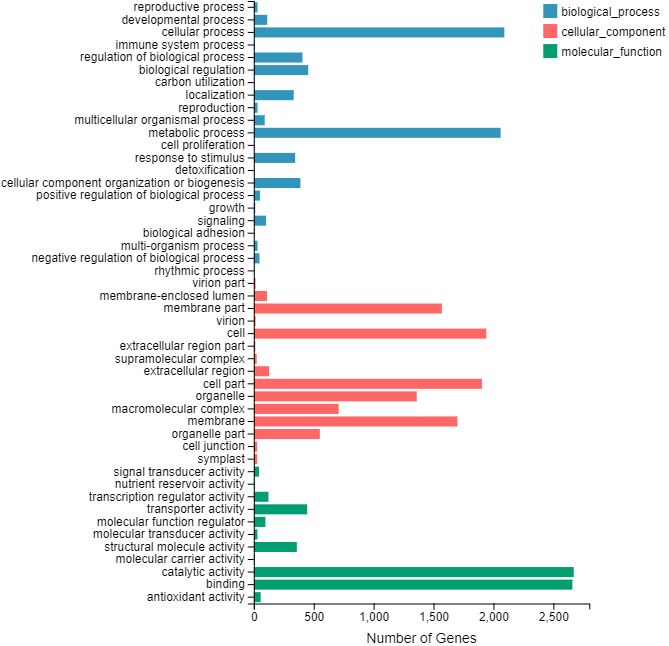

Supplement: Figure S9 [file peerj-08-10157-s009.png]
